# Psychological mechanisms of work responsibility among Chinese university administrative staff: the roles of organizational identification, burnout, and role conflict

**DOI:** 10.3389/fpsyg.2026.1713808

**Published:** 2026-06-25

**Authors:** Xiao Lin, Chia Ching Tu

**Affiliations:** Department of Educational Administration, International College, Krirk University, Bangkok, Thailand

**Keywords:** job burnout, job responsibility, organizational identification, role conflict, service quality, sustainable development, university administrative staff

## Abstract

This study examined the psychological mechanisms underlying job responsibility among administrative staff in Chinese universities by integrating organizational identification, job burnout, and role conflict into a unified framework. Based on survey data from 438 administrative staff members at universities in southwestern China, structural equation modeling was used to test the proposed relationships. The results showed that organizational identification positively predicted job responsibility, whereas job burnout was negatively associated with job responsibility and partially mediated the relationship between organizational identification and job responsibility. Role conflict further moderated the relationship between job burnout and job responsibility, with burnout exerting a stronger negative effect under conditions of high role conflict. This study further extends the conceptualization of job responsibility by treating it as a responsibility-related behavioral orientation rather than merely a stable attitudinal trait. It also proposes a motivation–resource–context mechanism in which organizational identification provides motivational grounding, job burnout reflects psychological resource depletion, and role conflict represents contextual pressure. These findings extend research on responsibility-related behavior and offer practical implications for improving governance and service quality in higher education institutions.

## 1 Introduction

With the continual expansion of international student mobility and the growth of the cross-border education market, competition among higher education institutions has intensified (Altbach and Knight, 2007; Demange et al., 2020). Such institutions are knowledge-intensive service organizations, and the quality of services they provide not only affects student satisfaction (Butt et al., 2024; Surya Bahadur et al., 2024) but also influences institutional reputation and public evaluation (Alessandri et al., 2006; Fombrun, 1996). Accordingly, competition in higher education has gradually shifted from a model dominated by research output and academic reputation to a more comprehensive framework centered on service quality and governance capacity. This shift has been accompanied by the institutionalization of quality assurance systems that focus on learning outcomes; such systems have been implemented through accreditation standards and periodic institutional evaluations [Association to Advance Collegiate Schools of Business (Aacsb International, 2023; European Association for Quality Assurance in Higher Education [ENQA], 2015; Organisation for Economic Co-operation and Development [OECD], 2025; UNESCO, 2007)]. In China, this trend is reflected in the increasing emphasis on monitoring teaching quality and assessing learning outcomes; these efforts help promote continual improvement of quality governance systems (Yang, 2024). With the increasing refinement of institutional requirements, determining how to translate abstract quality standards into stable organizational practices has become a major concern in university governance.

As quality assurance requirements have become more institutionalized, university administrative staff have gradually assumed key roles in institutional implementation, process management, and organizational coordination (Gao, 2020; Yidana et al., 2023). In contrast to faculty members, who are primarily responsible for teaching and research, administrative staff must balance institutional rules and service provision, and they often face complex and multiple role demands. Therefore, the job performance of administrative staff depends not only on task completion but also on sustained engagement with their responsibilities and adherence to institutional norms. Accordingly, job responsibility constitutes a crucial psychological foundation linking institutional requirements to organizational practices (Gibbs and Kharouf, 2022; Veles et al., 2023).

At the organizational level, administrative staff members’ job responsibility plays a crucial role in improving service quality and organizational performance. Service quality theory states that employees’ sense of responsibility and service commitment directly influence their service delivery and stakeholder satisfaction (Grönroos, 1984; Liao and Chuang, 2004). At the national level, governance capacity and service quality in higher education have become crucial components of national competitiveness and soft power (Altbach, 2013). In practice, failure to actively cultivate and support administrative staff’s sense of responsibility may increase the likelihood of job burnout and undermine organizational effectiveness (Corbeanu et al., 2023; Maslach et al., 2001).

Research conducted in Western countries has provided valuable insights into administrative staff management in higher education (Gornitzka and Larsen, 2004; Veles et al., 2023; Whitchurch, 2008). However, few studies have systematically analyzed the mechanisms underlying responsibility-related behavior among administrative staff in Chinese higher education, where institutions are undergoing rapid transformation while also operating under mission-oriented governance (Green, 2023). Studies have suggested that employees’ behavior is influenced by the combined effects of psychological resources, emotional experiences, and role-related environments (Bakker and Demerouti, 2017; Bakker et al., 2023; Weiss and Cropanzano, 1996). The literature also indicates that organizational identification, which is a positive identity-based resource, enhances individuals’ sense of belonging and responsibility, thereby promoting their internalization of organizational goals and proactive role fulfillment (Ashforth and Mael, 1989; Riketta, 2005). By contrast, job burnout, a negative psychological state resulting from prolonged work stress, depletes an individual’s energy and impairs their self-regulation capacity, thereby constraining their performance (Karatuna et al., 2025; Lei et al., 2023). Role conflict, a typical situational stressor, also disrupts role performance by intensifying resource depletion (Corbeanu et al., 2023; Kahn et al., 1964). In the Chinese cultural context, which is characterized by a strong collectivist orientation, long-term organizational interactions tend to strengthen organizational identification (Tajfel and Turner, 1979; Zhang et al., 2022). Furthermore, hierarchical structures and relational norms such as the concept of “face” (i.e., concerns about social image and maintaining interpersonal harmony) may intensify role-related pressures (Hwang, 1987; Scaffidi Abbate et al., 2025), thereby amplifying the combined effects of the aforementioned psychological mechanisms.

Although studies have explored employee attitudes and behavioral outcomes from the perspectives of organizational identification, job burnout, and role conflict (Chen et al., 2025; Hu, 2024), several limitations remain. First, studies have predominantly focused on specific variables; consequently, the literature is fragmented and does not clearly explain how these psychological mechanisms interact (Mukherjee and Saritha, 2024; Schneider et al., 2025). In particular, although studies have established a direct relationship between organizational identification and employee behavior (Riketta, 2005), limited attention has been paid to how this relationship translates into concrete behavior under psychological resource constraints and situational pressures (Rovetta et al., 2025). Second, job responsibility is typically regarded in the literature as an expression of role obligation or attitudinal commitment (Fuller et al., 2006; Morrison and Phelps, 1999; Morrison, 2023; Pearce and Gregersen, 1991), and its formation as a behavioral orientation remains underexplored (Mukherjee and Saritha, 2024). Furthermore, research on higher education has primarily focused on faculty members or students, and relatively little attention has been paid to administrative staff, who are a key supporting workforce (Veles et al., 2023).

Addressing the aforementioned knowledge gaps is crucial. From a theoretical perspective, the lack of an integrative approach to examining several psychological mechanisms makes it difficult to understand how these mechanisms jointly influence responsibility-related behavior. From a practical perspective, administrative staff members’ sense of job responsibility directly affects their service quality and implementation of institutional policies and practices; neglecting to cultivate and support this sense of responsibility may increase the likelihood of job burnout and negatively affect organizational effectiveness (Corbeanu et al., 2023; Grönroos, 1984; Liao and Chuang, 2004; Maslach et al., 2001). Accordingly, to address these gaps, this study developed and empirically tested a theoretical framework integrating organizational identification, job burnout, and role conflict in the context of Chinese universities. The objective of the study was to explain how administrative staff members’ sense of job responsibility is shaped by motivational, resource-related, and contextual conditions and to provide insights that can serve as a reference for research in various institutional and cultural contexts.

## 2 Literature review

### 2.1 Organizational identification and job responsibility

Organizational identification theory states that when individuals incorporate their organizational membership into their self-concept and internalize organizational goals and values as part of their self-definition, they are more likely to understand their job responsibilities from the organization’s perspective and to regard fulfilling these responsibilities as an obligation (Ashforth and Mael, 1989; Mael and Ashforth, 1992). Hence, organizational identification affects individuals’ understanding of role meaning and facilitates the internalization of norms; consequently, external role requirements are transformed into internal behavioral standards, thereby providing a stable motivational foundation for responsibility-related behavior (Li et al., 2025).

Building on this theoretical basis, job responsibility is not only an attitudinal construct but also an action-oriented behavioral orientation (Jønsson and Fasano, 2025; Lan et al., 2025). In contrast to general work attitudes, job responsibility is demonstrated by individuals’ ability to translate their internalized sense of responsibility into sustained responsibility-related behavior. Therefore, organizational identification is crucial not merely because it strengthens individuals’ affective attachment to an organization but because it helps them incorporate organizational goals and role expectations into their self-concept, thereby increasing their intrinsic motivation to engage in responsibility-related behavior.

The work of university administrative staff is largely characterized by the implementation of institutional rules and procedures, coordination of processes, and continual provision of services (Veles et al., 2023). Hence, the development of responsibility-related behavior is influenced by both formal job requirements and whether staff members have internalized their organization’s goals and understand their responsibilities in terms of the organization’s overall interests (Wahl et al., 2025). In the Chinese cultural context, where collectivist values are prominent, individuals are likely to respond to organizational expectations and seek to maintain relational stability by fulfilling their role responsibilities. When tension arises between personal interests and organizational goals, a responsibility orientation that is derived from organizational identification tends to translate into sustained responsibility-related behavior (Chen et al., 2025). Accordingly, this study proposed the following hypothesis:

**H1**: Organizational identification has a significant positive effect on job responsibility among administrative staff in Chinese universities.

### 2.2 Mediating role of job burnout

Job burnout is a negative psychological state that results from prolonged work stress. It typically manifests as emotional exhaustion, depersonalization, and reduced personal accomplishment (Maslach and Jackson, 1981; Maslach et al., 2001; Maslach and Leiter, 2016; Zeng and Hu, 2024). Job burnout reflects the depletion of an individual’s emotional and cognitive resources in the presence of sustained work demands. That is, it represents high cumulative consumption of emotional and cognitive resources, which weakens an individual’s self-regulation capacity and reduces their willingness to maintain sustained effort, thereby affecting their ability to consistently fulfill their job responsibilities (Salvagioni et al., 2017). This indicates that job burnout serves both as a psychological response to prolonged stress and as a critical internal mechanism influencing responsibility-related behavior (Corbeanu et al., 2023).

According to organizational identification theory, when individuals incorporate organizational membership into their self-concept and internalize organizational goals and values as part of their self-definition, they are more likely to experience a sense of belonging and meaning (Ashforth et al., 2008; Haslam et al., 2009; Mael and Ashforth, 1992). The identity-based resource provided by organizational identification helps buffer individuals’ negative perceptions of work stress, thereby reducing the likelihood of job burnout (Haslam et al., 2009). Specifically, organizational identification provides a motivational foundation for responsibility-related behavior and also indirectly influences such behavior through its impact on individuals’ psychological resources.

The work of university administrative staff involves institutional implementation, process coordination, and multiparty collaboration; therefore, repetitive tasks and sustained responsibility demands jointly contribute to a high psychological workload (Lei et al., 2023; Pei et al., 2024). Research indicates that as job burnout accumulates, the emotional and cognitive resources required to sustain responsibility engagement are gradually depleted, thereby hindering the translation of organizational-identification-derived responsibility motivation into stable responsibility-related behavior (Magrone et al., 2024). This suggests that job burnout is a key psychological mechanism through which organizational identification affects job responsibility among university administrative staff. Accordingly, this study proposed the following hypothesis:

**H2**: Job burnout mediates the relationship between organizational identification and job responsibility among administrative staff in Chinese universities.

### 2.3 Moderating role of role conflict

Job burnout has a context-dependent effect on job responsibility, and the strength of this effect may depend on specific role environments. According to role conflict theory, when individuals perceive themselves to be subject to incompatible or difficult-to-reconcile role expectations in their workplace, role conflict becomes a major source of stress in role performance and increases psychological strain (Kahn et al., 1964; Rizzo et al., 1970). Under such conditions, individuals are required to invest additional effort to manage multiple demands and are more likely to experience a reduced capacity to cope with their work tasks because of inconsistencies among role expectations, which intensifies the negative effect of job burnout on job responsibility (Gilboa et al., 2008; Podsakoff et al., 2007).

The work of university administrative staff often requires them to switch frequently among teaching support, administrative management, service coordination, and external evaluation tasks; consequently, they tend to face diverse and occasionally inconsistent role expectations (Pitman, 2000). When the level of role conflict is high, individuals must respond to multiple role demands simultaneously, even if they are already experiencing job burnout. This further increases their emotional and cognitive burden and reduces their ability and willingness to sustain responsibility-related behavior. By contrast, when the level of role conflict is low, individuals retain greater flexibility in adjusting to changing demands, thereby mitigating the negative effect of job burnout on responsibility-related behavior (Gilboa et al., 2008; Nahrgang et al., 2011).

Role conflict primarily influences how psychological states translate into behavioral outcomes rather than how organizational identification is formed. Therefore, as a relatively stable form of self-concept integration, organizational identification is less directly affected by short-term situational pressures. By contrast, role conflict increases situational demands during the process of translating psychological states into behavioral outcomes, thereby intensifying the negative effect of job burnout on responsibility-related behavior. Hence, the present study conceptualized role conflict as a contextual moderating factor in the relationship between job burnout and job responsibility.

In the Chinese cultural context, which emphasizes maintaining relationships and interpersonal harmony, informal interaction norms (e.g., “face”) may make individuals more sensitive to others’ evaluations of their social image and to the stability of interpersonal relationships (Leung and Cohen, 2011; Zhou and Zhang, 2024). Therefore, university administrative staff members are more likely to comply with and assume tasks that are not included in their formal job requirements in order to maintain relationships, which intensifies situational pressure arising from role conflict (Chen and Chen, 2004; Zhao et al., 2020). In Chinese universities, role conflict can thus be considered both a major source of situational stress for administrative staff and a key boundary condition that influences the relationship between job burnout and job responsibility. Accordingly, this study proposed the following hypothesis:

**H3**: Role conflict moderates the relationship between job burnout and job responsibility among administrative staff in Chinese universities.

### 2.4 Conceptual framework

**FIGURE 1:**
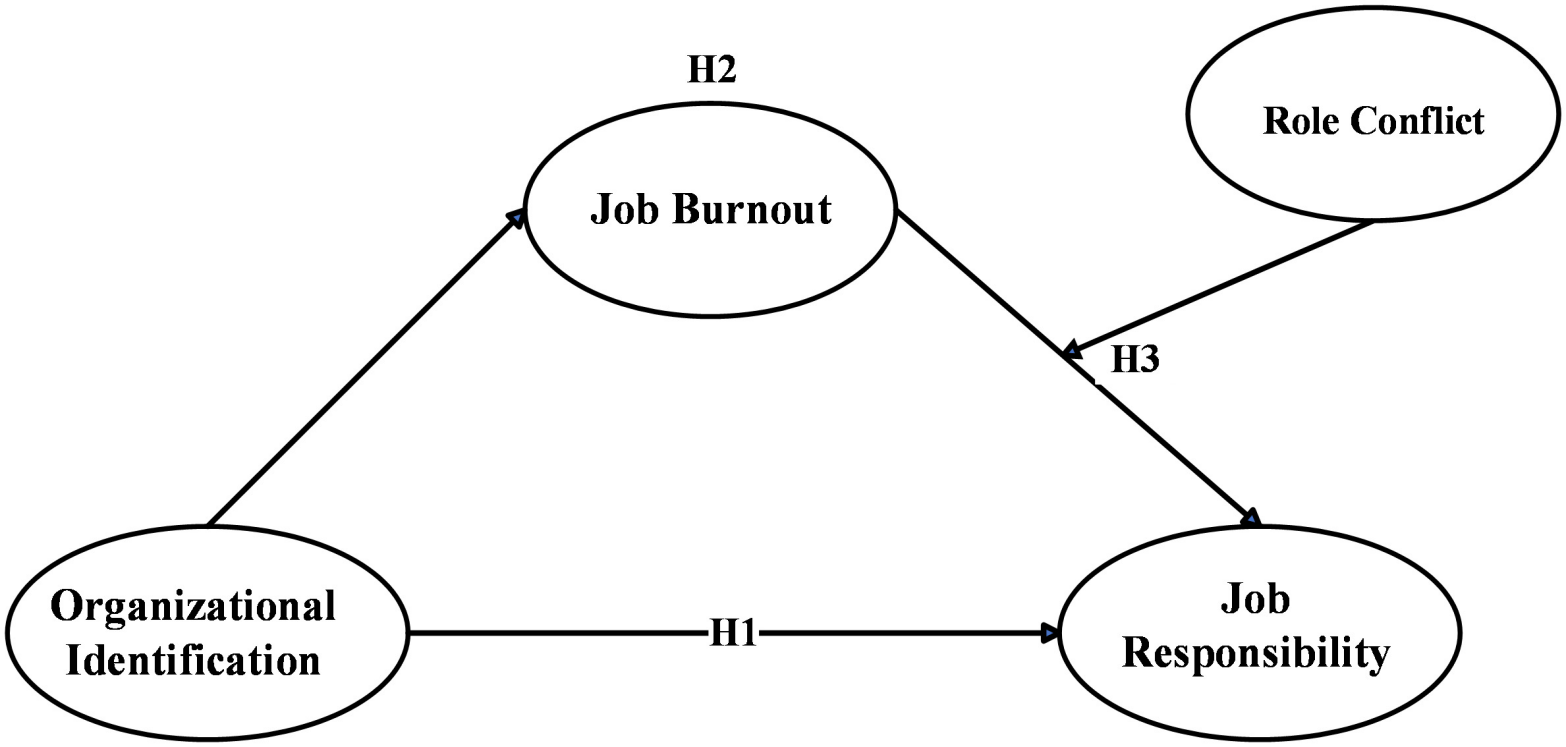
Model of proposed hypotheses.

On the basis of organizational identification theory, job burnout theory, and role conflict theory, this study developed an integrative conceptual framework focusing on the interaction between psychological state and work context to explain the formation of a sense of job responsibility among university administrative staff. In this framework, organizational identification was conceptualized as the motivational foundation of responsibility-related behavior. Job burnout was regarded as a mediating mechanism that reflected the state of individuals’ psychological resources and linked organizational identification to job responsibility. Role conflict was considered a contextual boundary condition that moderated the relationship between job burnout and job responsibility. This framework enabled the study to capture situational variations in the process through which psychological states are translated into behavioral outcomes. Figure 1 presents the conceptual model and the corresponding research hypotheses.

## 3 Materials and methods

### 3.1 Research design and sample

This study adopted a cross-sectional survey design to examine the relationships between various variables and their underlying psychological mechanisms. This design does not support the drawing of strict causal inferences (Maxwell and Cole, 2007; Podsakoff et al., 2003). Administrative staff members from universities in the Guangxi Zhuang Autonomous Region, southwestern China, were selected as respondents. This region was selected primarily because its higher education institutions operate under relatively constrained resource and support conditions (Dai et al., 2018), which reflect the broader context of limited resource investment in higher education institutions in western China. A total of 438 valid responses were obtained. Of the respondents, 194 were men (44.3%), and 244 were women (55.7%). The majority of the respondents were aged 30–40 years. These demographic characteristics are generally consistent with those previously reported for university staff in China.

### 3.2 Sampling strategy

Data were collected between May 7 and 27, 2024. A purposive sampling strategy was adopted to select respondents from eight universities. To ensure sample heterogeneity, these respondents were selected from universities that varied in terms of institutional level, type, and geographic location. This sampling approach is appropriate for testing theoretical mechanisms in a specific organizational setting (e.g., Kline, 2016). A structured questionnaire comprising 38 measurement items was used, and all items were rated on a 5-point Likert scale. Reverse-coded items were included to mitigate potential common method bias. The questionnaire was distributed online, and participation was voluntary and anonymous.

In structural equation modeling, the recommended sample size is generally 5–10 times the number of free parameters (Bentler and Chou, 1987). Accordingly, the final sample in the present study comprised 438 valid responses, which exceeded the recommended threshold and thus met the requirements for covariance-based structural equation modeling.

### 3.3 Data collection

Respondents were recruited through formal internal communication channels in the participating universities. Relevant administrative departments assisted in distributing the survey link to eligible administrative staff. The beginning of the questionnaire included an introduction outlining the purpose of the study. This introduction clearly stated that participation was entirely voluntary, that responses would be anonymously collected, and that the data obtained would be used only for academic research purposes and would remain strictly confidential. An individual proceeding with the survey after reading the introduction was considered to have indicated informed consent. The questionnaire was administered online through the Wenjuanxing platform (Ranxing Information Technology, Changsha, China). To encourage participation, individuals who completed the survey received a small symbolic monetary incentive (i.e., a nominal digital cash rewards, often referred to as a “red envelope” in Chinese contexts). This incentive was minimal and did not have a substantial influence on the respondents’ decision to participate.

### 3.4 Measures

This study used a structured questionnaire as its primary measurement instrument. Established scales with demonstrated reliability and validity were employed to measure the key constructs. Minor contextual adaptations were made to certain items to enhance their clarity and contextual relevance for university administrative staff in China while preserving the original theoretical meaning of the scales. To determine the measurement validity of these scales, confirmatory factor analysis (CFA) was conducted to assess the measurement models of all constructs in subsequent analyses.

#### 3.4.1 Job responsibility

Job responsibility was measured using the Chinese version of the work involvement scale applied by Hsieh (2017), which was based on the construct originally developed by Kanungo (1982). This scale assesses individuals’ psychological identification with and cognitive engagement in their work. The original scale is unidimensional and consists of nine items. Items with insufficient factor loadings (according to the CFA results) were removed. In the final scale, five items were retained for subsequent analysis (Table 1), and the scale exhibited favorable internal consistency reliability, with a Cronbach’s α of 0.861.

**TABLE 1:** Items for the job responsibility scale.

Item	Item description	Mean	Standard deviation	Factor loadings
Q10	I willingly work overtime when requested by my supervisor or university.	3.48	0.98	0.78
Q11	I sometimes neglect rest and meals when deeply involved in my work.	3.31	1.00	0.70
Q12	I voluntarily work extra hours to complete my tasks.	3.82	0.90	0.74
Q13	I arrive early to plan out my work for the day.	3.45	0.89	0.58
Q14	I strive to complete my work even without overtime compensation.	3.85	0.88	0.68

#### 3.4.2 Organizational identification

Organizational identification was measured using the scale developed by Mael and Ashforth (1992), which assesses the extent to which individuals define themselves in terms of their organizational membership. Four of the scale’s items were retained for subsequent analysis on the basis of the CFA results (Table 2). The scale demonstrated acceptable internal consistency reliability, with a Cronbach’s α of 0.776.

**TABLE 2:** Items for the organizational identification scale.

Item	Item description	Mean	Standard deviation	Factor loadings
Q6	When talking about my university, I refer to it as “my university” rather than using its name or saying “they.”	3.88	0.90	0.62
Q7	I perceive the progress of my university as a reflection of my own advancement.	3.79	0.90	0.78
Q8	I feel proud when others speak positively about my university.	3.95	0.86	0.83
Q9	I feel embarrassed when others criticize my university.	3.47	0.97	0.55

#### 3.4.3 Job burnout

Job burnout was measured using the Maslach Burnout Inventory–General Survey, which was developed by Schaufeli et al. (1996). This scale consists of three dimensions: emotional exhaustion, cynicism, and reduced personal accomplishment. In the present study, items measuring professional efficacy were reverse-coded before analysis to ensure consistency in the direction of all dimensions. On the basis of the CFA results, 14 items were retained for subsequent analysis (Table 3). The scale had high internal consistency reliability, with a Cronbach’s α of 0.920, and all subdimensions exhibited satisfactory reliability (emotional exhaustion: α = 0.923; depersonalization: α = 0.906; reduced personal accomplishment: α = 0.845).

**TABLE 3:** Items for the job burnout scale.

Item	Item description	Mean	Standard deviation	Factor loadings
Q15	My work leaves me physically and emotionally drained.	3.40	0.84	0.83
Q16	I feel completely exhausted at the end of the workday.	3.38	0.89	0.82
Q17	I find it hard to feel motivated when thinking about work in the morning.	2.95	1.03	0.84
Q18	The complexity of my work creates a great deal of stress.	3.34	0.93	0.87
Q19	I feel on the verge of burnout because of my job.	2.89	0.99	0.86
Q20	I am becoming less and less interested in my work.	2.88	1.03	0.86
Q21	I feel less motivated toward my job than I used to.	2.98	1.06	0.88
Q22	I question the meaning of my work.	2.67	1.14	0.85
Q23	I find myself emotionally distancing from my job.	2.37	1.01	0.77
Q24	I believe I have made meaningful contributions to my institution.	2.35	0.87	0.54
Q25	This job is a good fit for me.	2.50	0.95	0.74
Q26	Completing my work gives me a strong sense of accomplishment.	2.31	0.98	0.81
Q27	I have done a lot of meaningful work.	2.38	0.91	0.87
Q28	I can complete my tasks efficiently.	2.24	0.81	0.63

Q15–Q19 measure emotional exhaustion, Q20–Q23 measure depersonalization, and Q24–Q28 measure reduced personal accomplishment (reverse-coded).

#### 3.4.4 Role conflict

Role conflict was measured using the role conflict dimension from the Role Conflict and Role Ambiguity Scale developed by Rizzo et al. (1970). This scale has been widely used in organizational behavior and educational management research and has demonstrated good reliability and validity. In the present study, intrarole conflict and interrole conflict were not distinguished; they were instead treated as a single overall construct. On the basis of the CFA results, five items were retained for subsequent analysis (Table 4). Overall, the scale exhibited favorable internal consistency reliability, with a Cronbach’s α of 0.807.

**TABLE 4:** Items for the role conflict scale.

Item	Item description	Mean	Standard deviation	Factor loadings
Q1	I am often required to handle tasks in conflicting ways.	3.51	1.03	0.70
Q2	I am frequently asked to perform tasks unrelated to my core responsibilities.	3.48	1.12	0.82
Q3	Some of the tasks I am assigned lack adequate administrative resources.	3.13	0.99	0.60
Q4	My work requires collaboration with two or more administrative teams.	3.56	1.03	0.58
Q5	I receive assignments from two or more supervisors.	3.59	1.10	0.63

### 3.5 Data analysis

Data were analyzed using IBM SPSS Statistics (version 21) and IBM SPSS AMOS (version 24; IBM, Armonk, NY, USA). Because this study was conducted to test a theoretically derived latent variable model, covariance-based structural equation modeling was employed (Kline, 2016). This approach is appropriate for theory-driven research because it enables the simultaneous estimation of the complex relationships among latent variables and provides an overall evaluation of both measurement and structural models.

Descriptive statistics were used to summarize the demographic characteristics of the study sample. CFA was then conducted to assess the measurement model, including the factor loadings, reliability, and convergent validity of the constructs (Byrne, 2010). The heterotrait-to-monotrait ratio (HTMT) was used to further examine discriminant validity (Henseler et al., 2015).

After satisfactory model fit and measurement quality were established, the structural model was estimated to test the proposed hypotheses and examine the relationships between organizational identification, job burnout, role conflict, and job responsibility (Ahmed et al., 2024). Subsequently, the mediating role of job burnout in the relationship between organizational identification and job responsibility was tested using the bootstrap method, with 95% confidence intervals employed to assess the significance of indirect effects (MacKinnon, 2008).

A multigroup analysis was conducted to examine the moderating effect of role conflict on the relationship between job burnout and job responsibility (Byrne, 2010). The sample was divided into high- and low-role-conflict groups on the basis of role conflict scores, and between-group differences in key structural path coefficients were determined.

## 4 Results

### 4.1 Measurement model results

#### 4.1.1 CFA and measurement model fit

**FIGURE 2:**
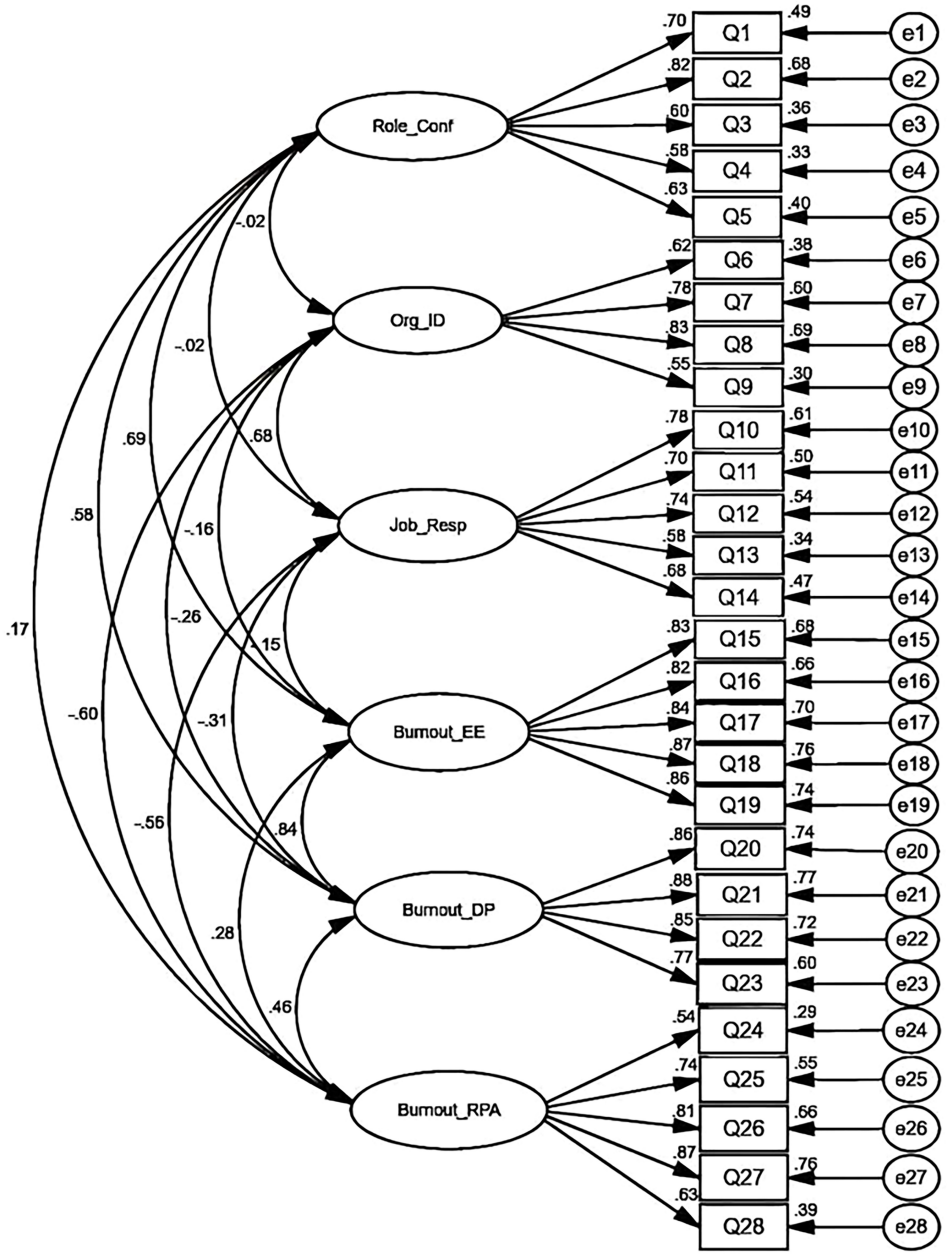
Results of confirmatory factor analysis for the measurement model. Role_Conf, role conflict; Org_ID, organizational identification; Job_Resp, job responsibility; Burnout_EE, emotional exhaustion; Burnout_DP, depersonalization; Burnout_RPA, reduced personal accomplishment.

Following standard structural equation modeling procedures, CFA was conducted to evaluate the measurement model, including the construct validity and measurement quality of the latent variables. A total of 28 measurement items across all constructs were retained in the final model (Figure 2 and Table 5).

**TABLE 5:** Reliability and validity of measurement model.

Indicator	Result/range
Univariate normality	Skewness: −1.072 to 0.511 Kurtosis: −0.731 to 1.775
Multivariate normality	Mardia’s coefficient = 212.332 < critical value 840
Standardized factor loadings	0.539–0.879
Model fit indices	χ^2^ = 1012.638, *p* < 0.001 χ^2^/df = 3.023 GFI = 0.855 CFI = 0.905 RMSEA = 0.068
Composite reliability (CR)	0.791–0.924
Average variance extracted (AVE)	0.452–0.710
Internal consistency	Cronbach’s α = 0.782–0.911
Common method bias	One-factor model: poor fit (χ^2^ = 3747.487, χ^2^/df = 10.707; GFI = 0.450; CFI = 0.522; RMSEA = 0.149)

CR, composite reliability; AVE, average variance extracted; GFI, goodness-of-fit index; CFI, comparative fit index; RMSEA, root mean square error of approximation. AVE values slightly below 0.50 are acceptable if CR is above 0.60 (Fornell and Larcker, 1981).

Normality test results revealed that the univariate skewness values ranged from −1.072 to 0.511 and that the kurtosis values ranged from −0.731 to 1.775; all values were noted to be within acceptable ranges. Regarding multivariate normality, Mardia’s coefficient was 212.332, which is below the critical value of *p*(*p* + 2) = 840 (*p* = 28); this finding suggests that the data did not significantly deviate from multivariate normality (Raykov and Marcoulides, 2008).

All latent variables were incorporated into a single measurement model, and the results indicated a good overall model fit (χ^2^ = 1012.638, *p* < 0.001, χ^2^/df = 3.023), with a goodness-of-fit index (GFI) of 0.855, a comparative fit index (CFI) of 0.905, and a root mean square error of approximation (RMSEA) of 0.068. All fit indices met the commonly recommended thresholds for structural equation modeling.

#### 4.1.2 Reliability and convergent validity

To further assess the quality of the measurement model, the reliability and convergent validity of the latent constructs were examined (Table 5). The results revealed that all standardized factor loadings were significant and exceeded the recommended threshold of 0.50. The composite reliability values for all constructs were higher than 0.70, indicating high internal consistency. Although the average variance extracted (AVE) values for role conflict (AVE = 0.452) and organizational identification (AVE = 0.493) were slightly below the conventional threshold of 0.50, they were still considered acceptable because of the relatively high composite reliability (Fornell and Larcker, 1981). Additionally, the Cronbach’s α coefficients for all constructs exceeded 0.70 (range: 0.782–0.911), further supporting the reliability of the measurement scales.

#### 4.1.3 Discriminant validity

The HTMT was used to assess discriminant validity among the constructs. The results revealed that the HTMT values ranged from 0.152 to 0.706, with all below the recommended threshold of 0.90, signifying satisfactory discriminant validity among the constructs.

#### 4.1.4 Common method bias

A single-factor model was constructed and tested to examine the potential impact of common method bias. The results indicated poor model fit (χ^2^ = 3747.487, χ^2^/df = 10.707), with a GFI of 0.450, a CFI of 0.522, and an RMSEA of 0.149, suggesting that a single factor did not account for the majority of the variance (Podsakoff et al., 2003). These findings demonstrated that common method bias was unlikely to pose a serious threat to the study results. Overall, the measurement model exhibited adequate reliability, convergent validity, and discriminant validity, indicating its suitability for subsequent structural model analysis.

### 4.2 Structural model and hypothesis testing

Once satisfactory model fit and measurement quality had been established, the structural model was estimated to test the proposed hypotheses.

#### 4.2.1 Direct effects of organizational identification

Path analysis revealed that organizational identification had a significant positive effect on job responsibility among university administrative staff (β = 0.65, *t* = 5.12, *p* < 0.001). Therefore, H1 was supported. Moreover, organizational identification had a significant negative effect on job burnout (β = −0.42, *t* = −4.36, *p* < 0.001), whereas job burnout had a significant negative effect on job responsibility (β = −0.34, *t* = −3.98, *p* < 0.001).

#### 4.2.2 Mediating effect of job burnout

Because the main structural paths were significant, the mediating effect of job burnout was further examined using the bootstrap method (MacKinnon, 2008). The overall model fit indices were as follows: χ^2^ = 952.432, *p* < 0.001, χ^2^/df = 4.252, GFI = 0.840, CFI = 0.883, and RMSEA = 0.086. The bootstrap results indicated that organizational identification exerted a significant indirect effect on job responsibility through job burnout (β = 0.08, *p* < 0.01). Furthermore, the direct effect of organizational identification on job responsibility remained significant (β = 0.65, *p* < 0.001). Overall, these findings demonstrated that job burnout partially mediated the relationship between organizational identification and job responsibility, which supported H2.

#### 4.2.3 Moderating effect of role conflict

A multigroup analysis was conducted to examine the moderating effect of role conflict. On the basis of the median value of the role conflict scores, the sample was divided into a high-role-conflict group (*n* = 189) and a low-role-conflict group (*n* = 249). The fit indices of the unconstrained model were as follows: χ^2^ = 1271.189, *p* < 0.001, χ^2^/df = 2.837, GFI = 0.803, CFI = 0.863, and RMSEA = 0.065.

Path analysis results revealed that job burnout had a significant negative effect on job responsibility in the high-role-conflict group (β = −0.25, *p* < 0.001) but a substantially weaker effect in the low-role-conflict group (β = −0.082). Further invariance testing of path coefficients (Byrne, 2010) demonstrated a significant difference between the unconstrained and constrained models [Δχ^2^(1) = 6.70, *p* < 0.05]. Overall, these findings suggest that role conflict significantly moderated the relationship between job burnout and job responsibility. Hence, H3 was supported.

## 5 Discussion

This study revealed that organizational identification contributes to the formation of job responsibility among administrative staff in Chinese universities. This finding is broadly consistent with those of previous research suggesting that organizational identification strengthens the alignment between individual behavior and organizational goals (Chen et al., 2025; van Knippenberg and van Schie, 2000). At the same time, the present study provides a more refined explanation at the mechanism level. Specifically, through the internalization of role norms, individuals can integrate organizational values and role expectations into their self-concept, and this integration enables them to gradually shift their responsibility-related behavior from context-dependent external responses to internally driven behavioral orientations. In contrast to a study that primarily interpreted this relationship from the perspective of behavioral alignment (Hubert et al., 2025), the present study further clarified the psychological mechanism through which organizational identification supports responsibility-related behavior.

The present study also extends the literature by shifting attention from general behavioral outcomes, such as organizational citizenship behavior and performance (Haslam et al., 2009), to job responsibility as a behavioral orientation. Organizational identification strengthens the consistency between individual behavior and organizational goals and stabilizes individuals’ internal behavioral standards, thereby supporting the sustained expression of responsibility-related behavioral engagement. This mechanism is particularly notable in collectivist cultural contexts and in the administrative setting of higher education institutions. In such contexts, individuals are more likely to incorporate organizational goals into their self-definition, thereby reinforcing the depth and stability of identity internalization (Hofstede, 2001; Zhang et al., 2022). In addition, compared with organizational contexts characterized by clearly defined responsibilities and specialized roles (Gornitzka and Larsen, 2004; Whitchurch, 2008), administrative positions in universities rely more heavily on sustained responsibility engagement, making the internalization of role norms a crucial condition for maintaining responsibility-related behavior.

Although organizational identification provides a major motivational foundation for responsibility-related behavior, this motivation does not necessarily translate into actual behavior and may be constrained by individuals’ psychological resource conditions. The present study revealed that job burnout plays a critical constraining role in the relationship between organizational identification and job responsibility. This finding is consistent with those of previous studies that have conceptualized job burnout as an essential mechanism linking stress to behavioral outcomes (Magrone et al., 2024; Maslach et al., 2001); the finding also indicates that the translation of motivation into behavior depends on the availability of psychological resources. Specifically, job burnout reflects the depletion of individuals’ emotional and cognitive resources, which weakens their self-regulation capacity and limits their ability to remain engaged in responsibility-related behavior (Brotheridge and Lee, 2002). A previous study also demonstrated that behavioral performance is closely associated with individuals’ psychological resource conditions (Sonnentag et al., 2017). The present findings further indicate that the process through which motivation is translated into behavior is systematically influenced by resource conditions.

From a mechanistic perspective, this study conceptualized responsibility-related behavior as the result of the joint operation of motivational sources and behavioral realization conditions. In this framework, job burnout constrains the relationship between motivation and behavioral performance by limiting psychological resources. Although previous research emphasized the motivational function of organizational identification (Ashforth et al., 2008), the present study proposes a motivation–resource mechanism highlighting the systematic role of individuals’ resource states in the process through which organizational identification influences responsibility-related behavior. This mechanism-based perspective helps explain why individuals with stronger organizational identification do not necessarily exhibit higher levels of behavioral engagement (Ashforth et al., 2008; Weisman et al., 2023); the perspective also extends the explanatory scope of organizational identification theory by integrating insights from job burnout theory and the psychological resource perspective.

In administrative work in Chinese universities, the identified mechanism has a clear practical basis. Administrative positions are characterized by coordination, service provision, and organizational support, and performance in these positions is often reflected in the level of responsibility engagement (Gornitzka and Larsen, 2004; Whitchurch, 2008). However, compared with teaching and research positions, administrative positions are less easily evaluated through quantifiable performance indicators, and career development opportunities are often constrained by evaluation systems that prioritize teaching and research (Shi, 2024). Under such institutional conditions, administrative staff are more likely to experience psychological resource depletion, which may intensify job burnout and strengthen its constraining effect on the translation of motivation into behavior.

The present study further found that although institutional constraints may be associated with job burnout, the role of organizational identification is not completely lost under such conditions. Previous studies have suggested that perceived organizational support, including institutional guarantees, career development opportunities, and interpersonal support, helps maintain a basic level of psychological engagement and buffers resource depletion under pressure (Eisenberger et al., 1986; Rhoades and Eisenberger, 2002). This buffering logic helps explain the findings of the present study in the administrative context of Chinese universities: even when administrative staff face relatively high levels of pressure, organizational support may provide conditions under which organizational identification continues to function. Therefore, job burnout does not fully account for the relationship between organizational identification and job responsibility. Accordingly, the present study incorporated the buffering logic of organizational support into the explanation of responsibility-related behavior formation, further clarifying why the role of organizational identification may be preserved under resource-constrained conditions.

In the present study, the effect of job burnout on responsibility-related behavior does not operate uniformly across contexts but is instead conditioned by the level of role conflict perceived by administrative staff. According to role conflict theory, multiple and inconsistent role expectations increase psychological pressure and influence individual behavior (Kahn et al., 1964; Rizzo et al., 1970). The results of this study also indicate that role conflict strengthens the negative effect of job burnout on responsibility-related behavior. This finding is consistent with those of previous research on role stress and resource depletion, suggesting that role conflict consumes individuals’ limited psychological resources and intensifies the influence of negative psychological states on behavioral performance (Gilboa et al., 2008; Nahrgang et al., 2011). In particular, this moderating effect is primarily observed in the relationship between job burnout and responsibility-related behavior, rather than operating throughout the entire process. Accordingly, role conflict can be understood as a contextual amplification mechanism; this indicates that role conflict serves as a key boundary condition in the process through which psychological states are translated into behavioral outcomes.

This contextual amplification mechanism is embedded in the institutional and cultural context of administrative work in Chinese universities. The hierarchical structure of university administrative systems makes it difficult for individuals to renegotiate the boundaries of their role when they must respond to demands from several levels and departments (Hofstede, 2001; Ruan et al., 2024), thereby increasing role conflict. Furthermore, relational norms such as “face” may increase individuals’ sensitivity to social evaluation, making them more likely to accept tasks beyond their formal job responsibilities in order to maintain organizational relationships and personal image (Chen and Chen, 2004; Hwang, 1987; Zhao et al., 2020; Zhou and Zhang, 2024). Under these combined influences, role conflict intensifies psychological resource depletion and strengthens the impact of job burnout on responsibility-related behavior.

In summary, this study adopted an integrative perspective combining psychological mechanisms and contextual boundary conditions to explain the mechanism through which responsibility-related behavior is formed among administrative staff in Chinese universities. Organizational identification provides a motivational foundation, job burnout constitutes a resource constraint, and role conflict strengthens the effect of resource depletion; together, these three elements influence the expression of responsibility-related behavior. By integrating these elements, the present study extends the literature and provides a more systematic and process-oriented explanation of the mechanisms through which responsibility-related behavior is formed in complex organizational contexts.

## 6 Implications

### 6.1 Theoretical implications

Most studies have examined individual behavior in organizational contexts characterized by clear role boundaries and relatively stable institutional arrangements (Biddle, 1986; Lan et al., 2025; Organ, 1988). By contrast, the present study extends the analytical context to university administration, which is a setting characterized by high complexity and strong coordination demands. The findings suggest that in such contexts, the formation of responsibility-related behavior relies heavily on the combined effects of several psychological mechanisms, thereby enhancing the explanatory power of existing theories in complex organizational environments.

On the basis of this foundation, the present study refines the explanatory pathway of organizational identification in the domain of responsibility-related behavior. Previous research in this field has primarily linked organizational identification to general behavioral outcomes such as organizational citizenship behavior and performance (Luo et al., 2025; Moon and Lim, 2025; Riketta, 2005). However, the present study demonstrated that the key role of organizational identification is the internalization of role norms. Through this internalization, external role requirements are transformed into internal behavioral standards, which shift responsibility-related behavior from being context-driven to internally driven and ensure that this behavior is sustained over time. This extends the theoretical value of organizational identification from behavioral alignment to behavioral sustainability.

The present study repositions the theoretical role of job burnout in the formation of responsibility-related behavior. In contrast to studies that have treated job burnout as an outcome of work-related stress (Maslach et al., 2001; Sonnentag et al., 2023), this study incorporated job burnout into the explanatory framework of behavior formation, highlighting its function as a psychological resource constraint. In this framework, job burnout constrains the translation of motivation into behavior by depleting psychological resources, which addresses a key gap in the understanding of why motivation does not necessarily lead to sustained behavioral engagement. Accordingly, this perspective enriches the application of job burnout theory in the explanation of behavioral processes.

The present study also provides a contextual extension of role conflict theory. Research has generally conceptualized role conflict as a source of work-related stress (Bakker and Demerouti, 2017). However, the present study demonstrated that role conflict limits individuals’ capacity for role adjustment in complex organizational contexts and that it intensifies the impact of resource depletion on behavioral outcomes. Accordingly, role conflict can be understood as a key contextual mechanism that influences responsibility-related behavior rather than merely as a source of work-related stress.

Overall, by integrating motivational sources, resource conditions, and contextual constraints, this study provides a comprehensive explanation for the formation of responsibility-related behavior. The proposed process framework, which integrates motivational sources, resource constraints, and contextual moderation, contributes to a more integrated application of organizational identification, job burnout, and role conflict theories in complex organizational settings.

### 6.2 Practical implications

From a managerial perspective, the sustainability of a sense of job responsibility among university administrative staff is not determined by isolated managerial practices. It requires the coordinated functioning of organizational support conditions and institutional arrangements. At the university governance level, merely strengthening performance requirements or increasing responsibility demands is unlikely to effectively enhance administrative staff’s responsibility engagement. Instead, universities should adopt institutional approaches that simultaneously enhance organizational identification, alleviate job burnout, and reduce role conflict.

Establishing clearer professional competency frameworks and differentiated evaluation systems and formally recognizing the professional contributions of administrative staff in university governance and operations are necessary to strengthen staff members’ organizational identification and sense of professional value (de Jong and del Junco, 2024; Vere et al., 2024). Furthermore, optimizing task allocation structures, reducing conflicting performance demands, and strengthening cross-departmental coordination mechanisms can help alleviate the pressure associated with multiple role expectations (Gibbs and Kharouf, 2022; Lee et al., 2024; Rizzo et al., 1970). These measures can mitigate the negative effects of job burnout on responsibility-related behavior and provide a stable human resource foundation for the sustained improvement of service quality and governance efficiency in higher education institutions.

In terms of institutional development, contemporary reforms in China aimed at increasing the professionalization of administrative staff through institutional and career development initiatives align with global trends in higher education, particularly the development of professional staff systems and dual career pathways (de Jong and del Junco, 2024; Whitchurch, 2015). Research has suggested that establishing independent and professionalized career development pathways for non-academic staff can enhance workforce stability and strengthen long-term responsibility engagement and organizational commitment (Muleya et al., 2022). The present findings provide practical insights into supporting responsibility-related behavior under complex institutional conditions and offer an empirically grounded perspective for comparative research on international higher education governance.

In summary, the sustainability of a sense of job responsibility among university administrative staff is determined by whether staff members’ organizational identification is effectively cultivated, their psychological resources are adequately maintained, and their role-related pressures are appropriately managed in a supportive institutional environment. When governance frameworks place greater emphasis on supportive conditions, role clarity, and professional recognition, administrative staff are more likely to sustain responsibility-related behavior in complex organizational contexts.

## 7 Conclusion

This study integrated organizational identification, job burnout, and role conflict into a unified analytical framework to systematically explain the psychological mechanisms underlying a sense of job responsibility among university administrative staff. The findings suggest that job responsibility among university administrative staff cannot be fully explained by a single psychological factor. Instead, it originates from the combined effects of responsibility motivation stimulated by organizational identification, psychological resource constraints reflected in job burnout, and situational pressures associated with role conflict. On the basis of these findings, this study suggests that responsibility-related behavior should be understood from a process-based perspective because the manifestation of such behavior depends on the interaction among motivation, resources, and contextual conditions rather than on a single attitudinal or institutional factor.

Overall, this study contributes to the understanding of the psychological foundations of responsibility-related behavior in the context of university administration from an integrative perspective. It also provides a novel explanatory pathway for the application of related theories in complex organizational environments.

## 8 Limitations and future research

Although this study provides a systematic explanation for the formation of a sense of job responsibility among university administrative staff, it has several limitations that should be acknowledged. First, the study employed cross-sectional data, which limits its ability to draw causal inferences. Future studies may employ longitudinal designs or experimental methods to examine the evolution of the relationships identified in this study. Second, data were collected using a self-report questionnaire. Although this approach is common in organizational research, future studies may incorporate multisource data or objective behavioral indicators to enhance the robustness of the findings. Finally, the study sample was drawn from a specific region in China. Therefore, the generalizability of the findings to other institutional and cultural contexts remains to be further examined. Future research may extend the present work through cross-regional or cross-national comparative studies.

## Data Availability

The datasets presented in this study can be found in online repositories. The names of the repository/repositories and accession number(s) can be found below: The datasets generated for this study can be found in the Open Science Framework (OSF): https://osf.io/mezqa/?view_only=956ad3d14543447f86fb9198bb053348.

## References

[B1] Aacsb International (2023). *2023 Standards for Business Accreditation.* Available online at: https://www.aacsb.edu/accreditation/standards/business (accessed January 9, 2026).

[B2] Ahmed R. R. Streimikiene D. Streimikis J. Siksnelyte-Butkiene I. (2024). A comparative analysis of multivariate approaches for data analysis in management sciences. *E&M Econ. Manage.* 27 192–210. 10.15240/tul/001/2024-5-001

[B3] Alessandri S. W. Yang S.-U. Kinsey D. F. (2006). An integrative approach to university visual identity and reputation. *Corporate Reput. Rev.* 9 258–270. 10.1057/palgrave.crr.1550033

[B4] Altbach P. G. (2013). *The International Imperative in Higher Education.* Rotterdam: Sense Publishers.

[B5] Altbach P. G. Knight J. (2007). The internationalization of higher education: motivations and realities. *J. Stud. Int. Educ.* 11 290–305. 10.1177/1028315307303542

[B6] Ashforth B. E. Mael F. (1989). Social identity theory and the organization. *Acad. Manage. Rev.* 14 20–39. 10.5465/amr.1989.4278999

[B7] Ashforth B. E. Harrison S. H. Corley K. G. (2008). Identification in organizations: an examination of four fundamental questions. *J. Manage.* 34 325–374. 10.1177/0149206308316059

[B8] Bakker A. B. Demerouti E. (2017). Job demands–resources theory: taking stock and looking forward. *J. Occupat. Health Psychol.* 22 273–285. 10.1037/ocp0000056 27732008

[B9] Bakker A. B. Demerouti E. Sanz-Vergel A. (2023). Job demands–resources theory: ten years later. *Annu. Rev. Organ. Psychol. Organ. Behav.* 10 25–53. 10.1146/annurev-orgpsych-120920-053933

[B10] Bentler P. M. Chou C.-P. (1987). Practical issues in structural modeling. *Sociol. Methods Res.* 16 78–117. 10.1177/0049124187016001004

[B11] Biddle B. J. (1986). Recent developments in role theory. *Annu. Rev. Sociol.* 12 67–92. 10.1146/annurev.so.12.080186.000435

[B12] Brotheridge C. M. Lee R. T. (2002). Testing a conservation of resources model of the dynamics of emotional labor. *J. Occup. Health Psychol.* 7 57–67. 11827234

[B13] Butt S. Umair T. Tajammal R. (2024). Nexus between key determinants of service quality and students’ satisfaction in higher education institutions (HEIs). *Ann. Hum. Soc. Sci.* 5 659–671. 10.35484/ahss.2024(5-II-S)62 42324378

[B14] Byrne B. M. (2010). *Structural Equation Modeling with AMOS: Basic Concepts, Applications, and Programming*, 2nd Edn. New York, NY: Routledge.

[B15] Chen K.-H. Hsieh M. H. M. Maritz A. Shieh C.-J. (2025). What affects organizational identification in universities? Examining the effects of psychological contract on organizational identification and commitment of teachers. *Acta Psychol.* 261:105756. 10.1016/j.actpsy.2025.105756 41177093

[B16] Chen X.-P. Chen C. C. (2004). On the intricacies of the Chinese guanxi: a process model of guanxi development. *Asia Pac. J. Manage.* 21 305–324. 10.1023/B:APJM.0000036465.19102.d5

[B17] Corbeanu A. Iliescu D. Ion A. Spînu R. (2023). The link between burnout and job performance: a meta-analysis. *Eur. J. Work Organ. Psychol.* 32 599–616. 10.1080/1359432X.2023.2209320

[B18] Dai Q. Ye X. Wei Y. D. Ning Y. Dai S. (2018). Geography, ethnicity and regional inequality in Guangxi Zhuang Autonomous Region, China. *Appl. Spatial Anal. Policy* 11 557–580. 10.1007/s12061-017-9229-3

[B19] de Jong S. del Junco C. (2024). How do professional staff influence academic knowledge development? A literature review and research agenda. *Stud. High. Educ.* 49 1042–1065. 10.1080/03075079.2023.2258155

[B20] Demange G. Fenge R. Uebelmesser S. (2020). Competition in the quality of higher education: the impact of student mobility. *Int. Tax Public Finance* 27 1224–1263. 10.1007/s10797-020-09595-5

[B21] Eisenberger R. Huntington R. Hutchison S. Sowa D. (1986). Perceived organizational support. *J. Appl. Psychol.* 71 500–507. 10.1037/0021-9010.71.3.500

[B22] European Association for Quality Assurance in Higher Education [ENQA] (2015). *Standards and Guidelines for Quality Assurance in the European Higher Education Area (ESG 2015).* Brussels: ENQA.

[B23] Fombrun C. J. (1996). *Reputation: Realizing Value from the Corporate Image.* Boston, MA: Harvard Business School Press.

[B24] Fornell C. Larcker D. F. (1981). Evaluating structural equation models with unobservable variables and measurement error. *J. Market. Res.* 18 39–50. 10.2307/3151312

[B25] Fuller J. B. Marler L. E. Hester K. (2006). Promoting felt responsibility for constructive change and proactive behavior: exploring aspects of an elaborated model of work design. *J. Organ. Behav.* 27 1089–1120. 10.1002/job.408

[B26] Gao H. L. (2020). Understanding the impact of administrative service quality on satisfaction and loyalty towards university students. *High. Educ. Res.* 5 25–30. 10.11648/j.her.20200501.15

[B27] Gibbs T. Kharouf H. (2022). The value of co-operation: an examination of the work relationships of university professional services staff and consequences for service quality. *Stud. High. Educ.* 47 38–52. 10.1080/03075079.2020.1725878

[B28] Gilboa S. Shirom A. Fried Y. Cooper C. (2008). A meta-analysis of work demand stressors and job performance: examining main and moderating effects. *Pers. Psychol.* 61 227–271. 10.1111/j.1744-6570.2008.00113.x

[B29] Gornitzka Å Larsen I. M. (2004). Towards professionalisation? Restructuring of administrative work in universities. *High. Educ.* 47 455–471. 10.1023/B:HIGH.0000020870.06667.f1

[B30] Green B. J. (2023). *How China’s System of Higher Education Works: Pragmatic Instrumentalism, Centralized-Decentralization, and Rational Chaos.* London: Routledge. 10.4324/9781003282372

[B31] Grönroos C. (1984). A service quality model and its marketing implications. *Eur. J. Market.* 18 36–44. 10.1108/EUM0000000004784

[B32] Haslam S. A. Jetten J. Postmes T. Haslam C. (2009). Social identity, health and well-being: an emerging agenda for applied psychology. *Appl. Psychol.* 58 1–23. 10.1111/j.1464-0597.2008.00379.x

[B33] Henseler J. Ringle C. M. Sarstedt M. (2015). A new criterion for assessing discriminant validity in variance-based structural equation modeling. *J. Acad. Market. Sci.* 43 115–135. 10.1007/s11747-014-0403-8

[B34] Hofstede G. (2001). *Culture’s Consequences: Comparing Values, behaviors, Institutions and Organizations Across Nations*, 2nd Edn. Thousand Oaks, CA: Sage.

[B35] Hsieh Y. T. (2017). *The Investigation of Correlations between Job Involvement and Job Satisfaction of Nursing Staff in a Psychiatric Teaching Hospital.* Kaohsiung: National Sun Yat-sen University.

[B36] Hu T. (2024). Balancing the equation: assessing the impact of management practices on staff and faculty wellbeing in Chinese higher education institutions. *Front. Psychol.* 15:1385612. 10.3389/fpsyg.2024.1385612 38882519 PMC11177615

[B37] Hubert P. Abdel Hadi S. Roswag M. Mojzisch A. Häusser J. A. (2025). Organizational identification moderates the effects of perceived COVID-19 safety climate on COVID-19 safety behavior in employees’ personal life: a social identity approach. *Appl. Psychol.* 17:e70057. 10.1111/aphw.70057 40611448 PMC12231930

[B38] Hwang K.-K. (1987). Face and favor: the Chinese power game. *Am. J. Sociol.* 92 944–974. 10.1086/228588 33955459 PMC8144941

[B39] Jønsson T. F. Fasano M. C. (2025). Theorizing subjective responsibility at work: an agentic approach. *Front. Psychol.* 16:1548931. 10.3389/fpsyg.2025.1548931 40709228 PMC12287070

[B40] Kahn R. L. Wolfe D. M. Quinn R. P. Snoek J. D. Rosenthal R. A. (1964). *Organizational Stress: Studies in Role Conflict and Ambiguity.* Hoboken, NJ: John Wiley & Sons.

[B41] Kanungo R. N. (1982). Measurement of job and work involvement. *J. Appl. Psychol.* 67 341–349. 10.1037/0021-9010.67.3.341

[B42] Karatuna I. Jönsson S. Dollard M. F. Muhonen T. (2025). The associations of the psychosocial safety climate with human service workers’ job demands, resources, and work- and health-related outcomes: a scoping review. *Safety Sci.* 191:106949. 10.1016/j.ssci.2025.106949

[B43] Kline R. B. (2016). *Principles and Practice of Structural Equation Modeling*, 4th Edn. New York, NY: Guilfordress.

[B44] Lan M. Hu Z. Nie T. (2025). Unwilling or unable? The impact of role clarity and job competence on frontline employees’ taking charge behaviors in hospitality industry. *Behav. Sci.* 15:526. 10.3390/bs15040526 40282148 PMC12024181

[B45] Lee M. C. C. Sim B. Y. H. Tuckey M. R. (2024). Comparing effects of toxic leadership and team social support on job insecurity, role ambiguity, work engagement, and job performance: a multilevel mediational perspective. *Asia Pac. Manage. Rev.* 29 115–126. 10.1016/j.apmrv.2023.09.002

[B46] Lei M. Alam G. M. Hassan A. B. (2023). Job burnout amongst university administrative staff members in China—a perspective on sustainable development goals (SDGs). *Sustainability* 15 8873. 10.3390/su15118873

[B47] Leung A. K. Y. Cohen D. (2011). Within- and between-culture variation: individual differences and the cultural logics of honor, face, and dignity. *J. Pers. Soc. Psychol.* 100 507–526. 10.1037/a0022151 21244179

[B48] Li J. Kaltiainen J. Hakanen J. J. (2025). How career crafting promotes employee well-being: the role of professional and organizational identification. *J. Bus. Psychol.* [Epub ahead of print]. 10.1007/s10869-025-10037-4

[B49] Liao H. Chuang A. (2004). A multilevel investigation of factors influencing employee service performance and customer outcomes. *Acad. Manage. J.* 47 41–58. 10.2307/20159559

[B50] Luo J. Ng L.-P. Choong Y.-O. (2025). The effect of responsible leadership on organizational citizenship behavior: double mediation of gratitude and organizational identification. *BMC Psychol.* 13:6. 10.1186/s40359-024-02337-w 39754288 PMC11699642

[B51] MacKinnon D. P. (2008). *Introduction to Statistical Mediation Analysis.* New York, NY: Lawrence Erlbaum Associates.

[B52] Mael F. A. Ashforth B. E. (1992). Alumni and their alma mater: a partial test of the reformulated model of organizational identification. *J. Organ. Behav.* 13 103–123. 10.1002/job.4030130202

[B53] Magrone M. Montani F. Emili S. Bakker A. B. Sommovigo V. (2024). A new look at job demands, resources, and volunteers’ intentions to leave: the role of work–home interference and burnout. *Voluntas* 35 1118–1130. 10.1007/s11266-024-00679-y

[B54] Maslach C. Jackson S. E. (1981). The measurement of experienced burnout. *J. Organ. Behav.* 2 99–113. 10.1002/job.4030020205

[B55] Maslach C. Leiter M. P. (2016). Understanding the burnout experience: recent research and its implications for psychiatry. *World Psychiatry* 15 103–111. 10.1002/wps.20311 27265691 PMC4911781

[B56] Maslach C. Schaufeli W. B. Leiter M. P. (2001). Job burnout. *Annu. Rev. Psychol.* 52 397–422. 10.1146/annurev.psych.52.1.397 11148311

[B57] Maxwell S. E. Cole D. A. (2007). Bias in cross-sectional analyses of longitudinal mediation. *Psychol. Methods* 12 23–44. 10.1037/1082-989X.12.1.23 17402810

[B58] Moon K.-K. Lim J. (2025). Exploring the impact of organizational identification on innovative work behavior in the Korean public sector: the moderating role of charismatic leadership. *Behav. Sci.* 15:1218. 10.3390/bs15091218 41009248 PMC12466728

[B59] Morrison E. W. (2023). Employee voice and silence: taking stock a decade later. *Annu. Rev. Organ. Psychol. Organ. Behav.* 10 79–107. 10.1146/annurev-orgpsych-120920-054654

[B60] Morrison E. W. Phelps C. C. (1999). Taking charge at work: extrarole efforts to initiate workplace change. *Acad. Manage. J.* 42 403–419. 10.2307/257011 23787629

[B61] Mukherjee U. Saritha S. R. (2024). Unethical pro-organizational behavior: a systematic literature review and research agenda. *Int. J. Ethics Syst.* 42 378–417. 10.1108/IJOES-11-2023-0243

[B62] Muleya D. Ngirande H. Terera S. R. (2022). The influence of training and career development opportunities on affective commitment: a South African higher education perspective. *SA J. Hum. Resour. Manage.* 20:a1620. 10.4102/sajhrm.v20i0.1620

[B63] Nahrgang J. D. Morgeson F. P. Hofmann D. A. (2011). Safety at work: a meta-analytic investigation of the link between job demands, job resources, burnout, engagement, and safety outcomes. *J. Appl. Psychol.* 96 71–94. 10.1037/a0021484 21171732

[B64] Organ D. W. (1988). *Organizational Citizenship Behavior: The Good Soldier Syndrome.* Lexington, MA: Lexington Books.

[B65] Organisation for Economic Co-operation and Development [OECD] (2025). *Ensuring Quality in Vocational Education and Training and Higher Education.* Paris: OECD Publishing.

[B66] Pearce J. L. Gregersen H. B. (1991). Task interdependence and extrarole behavior: a test of the mediating effects of felt responsibility. *J. Appl. Psychol.* 76 838–844. 10.1037/0021-9010.76.6.838

[B67] Pei S. Wang S. Jiang R. Guo J. Ni J. (2024). How work stress influences turnover intention among Chinese local undergraduate university teachers: the mediating effect of job burnout and the moderating effect of self-efficacy. *Front. Public Health* 12:1308486. 10.3389/fpubh.2024.1308486 38566801 PMC10985245

[B68] Pitman T. (2000). Perceptions of academics and students as customers: a survey of administrative staff in higher education. *J. High. Educ. Policy Manage.* 22 165–175. 10.1080/713678138

[B69] Podsakoff N. P. LePine J. A. LePine M. A. (2007). Differential challenge stressor–hindrance stressor relationships with job attitudes, turnover intentions, turnover, and withdrawal behavior: a meta-analysis. *J. Appl. Psychol.* 92 438–454. 10.1037/0021-9010.92.2.438 17371090

[B70] Podsakoff P. M. MacKenzie S. B. Lee J.-Y. Podsakoff N. P. (2003). Common method biases in behavioral research: a critical review of the literature and recommended remedies. *J. Appl. Psychol.* 88 879–903. 10.1037/0021-9010.88.5.879 14516251

[B71] Raykov T. Marcoulides G. A. (2008). *An Introduction to Applied Multivariate Analysis*. New York, NY: Routledge.

[B72] Rhoades L. Eisenberger R. (2002). Perceived organizational support: a review of the literature. *J. Appl. Psychol.* 87 698–714. 10.1037/0021-9010.87.4.698 12184574

[B73] Riketta M. (2005). Organizational identification: a meta-analysis. *J. Vocat. Behav.* 66 358–384. 10.1016/j.jvb.2004.05.005

[B74] Rizzo J. R. House R. J. Lirtzman S. I. (1970). Role conflict and ambiguity in complex organizations. *Admin. Sci. Q.* 15 150–163. 10.2307/2391486 31549049 PMC6750067

[B75] Rovetta A. Bortolotti A. Palumbo R. (2025). Integrating team and organizational identity: a systematic literature analysis. *Front. Organ. Psychol.* 2:1439269. 10.3389/forgp.2024.1439269

[B76] Ruan J. Cai Y. Stensaker B. (2024). University managers or institutional leaders? An exploration of top-level leadership in Chinese universities. *High. Educ.* 87 703–719. 10.1007/s10734-023-01031-x 37362758 PMC10088719

[B77] Salvagioni D. A. J. Melanda F. N. Mesas A. E. González A. D. Gabani F. L. Andrade S. M. (2017). Physical, psychological and occupational consequences of job burnout: a systematic review of prospective studies. *PLoS One* 12:e0185781. 10.1371/journal.pone.0185781 28977041 PMC5627926

[B78] Scaffidi Abbate C. Bonfanti R. C. Misuraca R. Ruggieri S. (2025). Power distance in the workplace and its effect on prosocial behavioral intentions. *Acta Psychol.* 253:104695. 10.1016/j.actpsy.2025.104695 39813943

[B79] Schaufeli W. B. Leiter M. P. Maslach C. Jackson S. E. (1996). “Maslach burnout inventory–general survey (MBI-GS),” in *Maslach Burnout Inventory manual*, 3rd Edn, eds Maslach C. Jackson S. E. Leiter M. P. (Palo Alto, CA: Consulting Psychologists Press), 19–26.

[B80] Schneider J. Kern M. Lorenz T. (2025). Unifying work values: establishing a circular framework based on basic human values. *Front. Psychol.* 16:1526799. 10.3389/fpsyg.2025.1526799 40861345 PMC12376437

[B81] Shi G. (2024). Research and practice on the development path of university administrative staff teams. *Int. J. Educ. Teach. Res.* 1:311. 10.70767/ijetr.v1i2.311

[B82] Sonnentag S. Tay L. Nesher Shoshan H. (2023). A review on health and well-being at work: more than stressors and strains. *Pers. Psychol.* 76 473–510.

[B83] Sonnentag S. Venz L. Casper A. (2017). Advances in recovery research: What have we learned? What should be done next? *J. Occup. Health Psychol.* 22 365–380. 10.1037/ocp0000079 28358572

[B84] Surya Bahadur G. C. Gurung S. K. Poudel R. L. Yadav U. S. Bhattacharjee A. Dhungana B. R. (2024). The effect of higher education service quality on satisfaction among business students in India and Nepal. *Cogent Educ.* 11:2393521. 10.1080/2331186X.2024.2393521

[B85] Tajfel H. Turner J. C. (1979). “An integrative theory of intergroup conflict,” in *The Social Psychology of Intergroup Relations*, eds Austin W. G. Worchel S. (Monterey, CA: Brooks/Cole), 33–47.

[B86] UNESCO (2007). *Quality Assurance and Accreditation: A Glossary of Basic Terms and Definitions.* Paris: UNESCO.

[B87] van Knippenberg D. van Schie E. C. M. (2000). Foci and correlates of organizational identification. *J. Occupat. Organ. Psychol.* 73 137–147. 10.1348/096317900166949

[B88] Veles N. Graham C. Ovaska C. (2023). University professional staff roles, identities, and spaces of interaction: systematic review of literature published in 2000–2020. *Policy Rev. High. Educ.* 7 127–168. 10.1080/23322969.2023.2193826

[B89] Vere K. Verney C. Webster-Deakin T. (2024). Crossing and dismantling boundaries: recognising the value of professional staff within higher education. *Lond. Rev. Educ.* 22:29. 10.14324/LRE.22.1.29

[B90] Wahl I. Einwiller S. A. Bartels J. (2025). Fostering employees’ organizational identification and organizational citizenship behavior through diversity communication. *Manage. Commun. Q.* 40 191–223. 10.1177/08933189251361810

[B91] Weisman H. Wu C.-H. Yoshikawa K. Lee H.-J. (2023). Antecedents of organizational identification: a review and agenda for future research. *J. Manage.* 49 2030–2061. 10.1177/01492063221140049

[B92] Weiss H. M. Cropanzano R. (1996). Affective events theory: a theoretical discussion of the structure, causes and consequences of affective experiences at work. *Res. Organ. Behav.* 18 1–74.

[B93] Whitchurch C. (2008). Shifting identities and blurring boundaries: the emergence of third space professionals in UK higher education. *High. Educ. Q.* 62 377–396. 10.1111/j.1468-2273.2008.00387.x

[B94] Whitchurch C. (2015). “The rise of third space professionals: paradoxes and dilemmas,” in *Forming, Recruiting, and Managing the Academic Profession*, eds Teichler U. Cummings W. K. (Dordrecht: Springer), 79–99.

[B95] Yang Y. (2024). Rethinking the challenges faced by the quality assurance system in Chinese higher education from the perspective of quality culture. *Educ. Rev. USA* 8 1015–1022. 10.26855/er.2024.08.001

[B96] Yidana P. Bangase E. A. Bagina R. Billa G. (2023). A model of administrative service quality in higher education. *Br. J. Educ. Learn. Dev. Psychol.* 6 52–75. 10.52589/BJELDP-XX8LIQLC

[B97] Zeng P. Hu X. (2024). A study of the psychological mechanisms of job burnout: implications of person–job fit and person–organization fit. *Front. Psychol.* 15:1351032. 10.3389/fpsyg.2024.1351032 39156820 PMC11328536

[B98] Zhang K. Wang Y. Tang N. (2022). Power distance orientation and perceived insider status in China: a social identity perspective. *Asia Pac. Bus. Rev.* 29 89–113. 10.1080/13602381.2022.2082115

[B99] Zhao Z. J. Chen H. H. Li K. W. (2020). Management of interpersonal conflict in negotiation with Chinese: a perceived face threat perspective. *Group Decis. Negotiat.* 29 75–102. 10.1007/s10726-019-09645-2

[B100] Zhou B. Zhang S. (2024). Exploring mianzi consciousness congruence and its impact on unethical pro-organizational behavior. *BMC Psychol.* 12:436. 10.1186/s40359-024-01934-z 39135140 PMC11320878

